# Adipose atrophy in cancer cachexia: morphologic and molecular analysis of adipose tissue in tumour-bearing mice

**DOI:** 10.1038/sj.bjc.6603360

**Published:** 2006-10-03

**Authors:** C Bing, S Russell, E Becket, M Pope, M J Tisdale, P Trayhurn, J R Jenkins

**Affiliations:** 1 Obesity Biology Unit, Division of Metabolic & Cellular Medicine, School of Clinical Sciences, University of Liverpool, Liverpool L69 3GA, UK; 2 Pharmaceutical Sciences Research Institute, Aston University, Birmingham, B4 7ET, UK; 3 Department of Veterinary Pathology, University of Liverpool, Liverpool L69 3BX, UK; 4 The Henry Wellcome Laboratory of Molecular and Cellular Gastroenterology, School of Clinical Sciences, University of Liverpool, Liverpool L69 3BX, UK

**Keywords:** cancer cachexia, adipose tissue, C/EBP*α*, SREBP-1c, mice

## Abstract

Extensive loss of adipose tissue is a hallmark of cancer cachexia but the cellular and molecular basis remains unclear. This study has examined morphologic and molecular characteristics of white adipose tissue in mice bearing a cachexia-inducing tumour, MAC16. Adipose tissue from tumour-bearing mice contained shrunken adipocytes that were heterogeneous in size. Increased fibrosis was evident by strong collagen-fibril staining in the tissue matrix. Ultrastructure of ‘slimmed’ adipocytes revealed severe delipidation and modifications in cell membrane conformation. There were major reductions in mRNA levels of adipogenic transcription factors including CCAAT/enhancer binding protein alpha (C/EBP*α*), CCAAT/enhancer binding protein beta, peroxisome proliferator-activated receptor gamma, and sterol regulatory element binding protein-1c (SREBP-1c) in adipose tissue, which was accompanied by reduced protein content of C/EBP*α* and SREBP-1. mRNA levels of SREBP-1c targets, fatty acid synthase, acetyl CoA carboxylase, stearoyl CoA desaturase 1 and glycerol-3-phosphate acyl transferase, also fell as did glucose transporter-4 and leptin. In contrast, mRNA levels of peroxisome proliferators-activated receptor gamma coactivator-1alpha and uncoupling protein-2 were increased in white fat of tumour-bearing mice. These results suggest that the tumour-induced impairment in the formation and lipid storing capacity of adipose tissue occurs in mice with cancer cachexia.

Cancer cachexia is a metabolic disorder characterised by progressive loss of body weight with depletion of skeletal muscle and adipose tissue (Argiles *et al*, 2003). Cachexia and its consequences are detrimental and considered to be the direct cause of ∼20% of cancer deaths (Tisdale, 2002). Adipose atrophy is a hallmark of cancer cachexia, up to a 85% decrease in body fat being reported in lung cancer patients, which may lead to hyperlipidaemia and insulin resistance as well as complicate antitumour therapies. Loss of fat stores cannot be explained by reduced appetite alone as it often precedes the onset of anorexia and is severe in animal model of cachexia than that of food restriction (Bing *et al*, 2000). Evidence has accumulated that the disease progress triggers catabolic responses that override anabolism at the peripheral tissues.

The importance of white adipose tissue in the control of adiposity has been recognised with the discovery of adipocyte-secreted adipokines which regulate body weight (Trayhurn & Bing, 2006), and by the studies indicating that adipocyte metabolism potently affects body fat accumulation (Orci *et al*, 2004). Although the defects in adipocyte development and metabolism are implicated in the pathogenesis of HIV-related lipodystrophy (Bastard *et al*, 2002), little is known the mechanism underlying malignancy-related lipoatrophy. Lipolysis appears to be increased in cancer cachexia since in white fat lipoprotein lipase activity is decreased in tumour-bearing mice (Thompson *et al*, 1981) while hormone sensitive lipase mRNA is elevated in cancer patients with cachexia (Thompson *et al*, 1993). In addition, hyperlipidaemia is often present in cachectic cancer patients (Rofe *et al*, 1994) and in tumour-bearing rats (Lopez-Soriano *et al*, 1997a). These changes are thought to be induced by cachexia mediators such as the inflammatory cytokine TNF*α* and tumour-derived lipid-mobilising factors (Tisdale, 2002).

Adipose tissue mass is also influenced by adipogenesis that involves the recruitment of new adipocytes (preadipocyte differentiation) and adipocyte maturation. Adipocyte differentiation is a highly controlled process through sequential activation of transcription factors that regulate the expression of adipocyte-specific markers (Mandrup & Lane, 1997). The early event involves increases in C/EBP*β* and *δ* in a transient manner, which enables the distinction between a preadipocyte and nonadipogenic precursor cell. C/EBP*β* and *δ* activate the expression of peroxisome proliferator-activated receptor gamma (PPAR*γ*), which in turn stimulates C/EBP*α* expression, and C/EBP*α* synergises with PPAR*γ* in controlling terminal differentiation (Rosen, 2005). Differentiation is enhanced by sterol regulatory element binding protein-1c (SREBP-1c), which activates PPAR*γ* transcription (Fajas *et al*, 1999). Adipocyte maturation is accompanied by intracellular accumulation of lipid, and SREBP-1c activates the lipogenic pathway by stimulating the expression of genes encoding lipogenic enzymes, such as acetyl-CoA carboxylase (ACC), fatty acid synthase (FAS), stearoyl-CoA desaturase-1 (SCD-1) and glycerol-3-phosphate acyltransferase (GPAT) (Shimano *et al*, 1999). Glucose, which serves as a substrate for lipid synthesis, is transported into the adipocyte via the insulin-responsive glucose transporter-4 (Glut-4). However, it remains unknown whether the tumour-burden affects adipocyte development in adipose tissue.

Peroxisome proliferators-activated receptor gamma coactivator-1*α* (PGC-1*α*) plays a central role in the coordination of adaptive thermogenesis through increased mitochondrial biogenesis and oxidative metabolisms in brown adipose tissue (BAT) and skeletal muscle (Puigserver *et al*, 1998). PGC-1*α* is also expressed in white fat with low baseline levels in both rodents and humans (Kakuma *et al*, 2000; Semple *et al*, 2004). Overexpression of PGC-1*α* in human adipocytes enhances mitochondrial activities prompting white adipocytes away from fat storage towards lipid utilisation (Tiraby *et al*, 2003). Conversely, diminished PGC-1*α* mRNA in white fat has been reported in morbidly obese subjects (Semple *et al*, 2004). A question that has not yet been explored is whether PGC-1*α* expression in white fat is altered in cancer cachexia.

In this study, we have examined the morphologic characteristics of white adipose tissue from mice bearing the MAC16 colon adeocarcinoma, a cachexia model which induces profound loss of lean mass and fat mass without severe anorexia (Bibby *et al*, 1987). We have further explored the molecular basis by which tumour-burden could alter adipose tissue mass and function, by assessing the expression of genes that control adipocyte differentiation, lipogenesis and lipid utilisation in white fat of tumour-bearing mice as compared with both freely fed and pair-fed nontumour-bearing controls. We show that the tumour-burden induces profound alterations of adipose tissue plasticity and ultrastructural modifications of adipocytes. Our data also show the major reductions in the transcription of adipogenic and lipogenic factors, suggesting that impairment in the formation and lipid-storing function of adipose tissue occurs in cancer cachexia.

## MATERIALS AND METHODS

### Animals

NMRI mice (20–22 g) from an inbred colony at Aston University (Birmingham, UK) were kept at an ambient temperature of 22±2°C under a 12 : 12 h light–dark cycle (lights on at 0700 hours) and fed a standard diet (SDS economy breeder; Lillico, UK) with water ad libitum. Fragments of the MAC16 tumour were implanted subcutaneously into the flank of mice using a trocar. The weight-matched mice underwent sham operation and served as freely fed or pair-fed controls, which were pair-fed to match the food intake of tumour-bearing mice. Food intake and body weight were recorded. At 18 days after tumour inoculation, mice were killed by cervical dislocation. Epididymal white fat and interscapular BAT were removed and were fixed for microscopy or snap-frozen in liquid N_2_ and then stored at −80°C until analysis. Animal studies were conducted according to the UKCCCR Guidelines for the care and use of laboratory animals.

### Light microscopy

Epididymal fat pads from tumour-bearing and control mice were fixed in 10% neutral formalin for 24 h, dehydrated in absolute ethanol, cleared in xylene and then embedded in paraffin. The paraffin was cut into 5-*μ*m sections that were stained with Harris haematoxylin, counterstained with eosin, and then evaluated by light microscopy. To detect collagen fibre content, sections were stained with Sirius Red. For quantification of adipocyte size, sections were analysed by using an Optiphot-2 microscope (Nikon, Japan) equipped with a digital camera. The cell perimeter and sectional area were measured in 100 adipocytes per section (three sections for one mouse and five animals per group), and data analysis performed using the MCID Basic software (Imaging Reseaech Inc., Ontario, Canada) for digital image processing.

### Electron microscopy

Epididymal fat pads were cut into slices while fixed in 2.5% glutaraldehyde and 4% paraformaldehyde in 0.1 M cacodylate buffer (pH 7.4). Specimens were postfixed with 1% osmium tetroxide, dehydrated through an ascending series of ethanol, and embedded in Taab epoxy resin. Semithin (2 *μ*m) and ultrathin sections (60 nm) were cut on a Reichert–Jung ultracut microtome. The semithin sections were stained with 1% toluidine blue and examined with a light microscope. The adjacent thin sections were double-contrasted with uranylacetate followed by lead citrate, and viewed and photographed with a Hitachi H600 transmission electron microscope (Hitachi Ltd, Tokyo, Japan).

### Real-time PCR

Total RNA was extracted from tissues using Trizol (Invitrogen, Carlsbad, CA, USA) and the RNA concentration determined from the absorbance at 260 nm. First strand DNA was reverse transcribed from 1 *μ*g of total RNA by using a Reverse-iT™ first strand synthesis kit (ABgene, Epson, UK) in a final volume of 20 *μ*l. The real-time PCR amplification was performed in a final volume of 25 *μ*l, containing 1 *μ*l cDNA (equivalent to 50 ng of RNA), forward and reverse primers, TaqMan®probe FAM-TAMRA (Table 1), and a master mix made from qPCR core kit (Eurogentec, Southampton, UK). PCR amplifications and efficiency were optimised according to the manufacturer's protocol and performed by using the ABI PRISM 7700 Sequence Detector (Applied Biosystems, Foster City, CA, USA) described previously (Bing *et al*, 2004). All samples were normalised to the *β*-actin values and the results were expressed as fold changes of *C*_t_ value relative to controls using the 
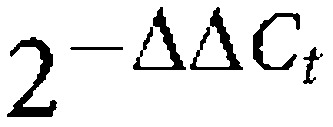
 formula (Livak & Schmittgen, 2001). *β*-Actin was used as a reference gene since we have experimentally determined, that there were no statistically significant differences of its *C*_t_ values between control and tumour-bearing or pair-feding groups (both *P*>0.05, three experiments).

### Western blotting

Proteins were isolated from epididymal fat. Samples containing 20 *μ*g of protein were separated by electrophoresis on a 12% SDS–polyacrylamide gel. Proteins were then transferred to nitrocellulose membranes (Amersham Inc., UK). Immunoblotting was performed using polyclonal antibodies against C/EBP*α* (14AA) and SREBP-1 (K-10) (Santa Cruz, CA, USA) at a 1 : 500 dilution. Blots were then incubated with sheep anti-rabbit IgG conjugated to horseradish peroxidase (Serotec, Oxford, UK) at 1 : 2000 for 1 h at room temperature, and were detected by enhanced chemiluminescence (ECL; Amersham, Buckinghamshire, UK). For the control of protein equal loading, blots were stripped and then incubated with a mouse monoclonal anti-*β*-tubulin antibody (Sigma, Poole, Dorset, UK) at 1 : 10 000, and subsequently incubated with a rabbit anti-mouse IgG (Santa Cruz) at 1 : 1000.

### Statistical analysis

Data are expressed as mean values ±s.e.m. Differences between groups were analysed by analysis of variance (ANOVA) coupled with Bonferroni's *t*-tests using ARCUS statistical software (Medical Computing, Liverpool, UK). Differences were considered as statistically significant when *P*<0.05.

## RESULTS

**Figure 1:**
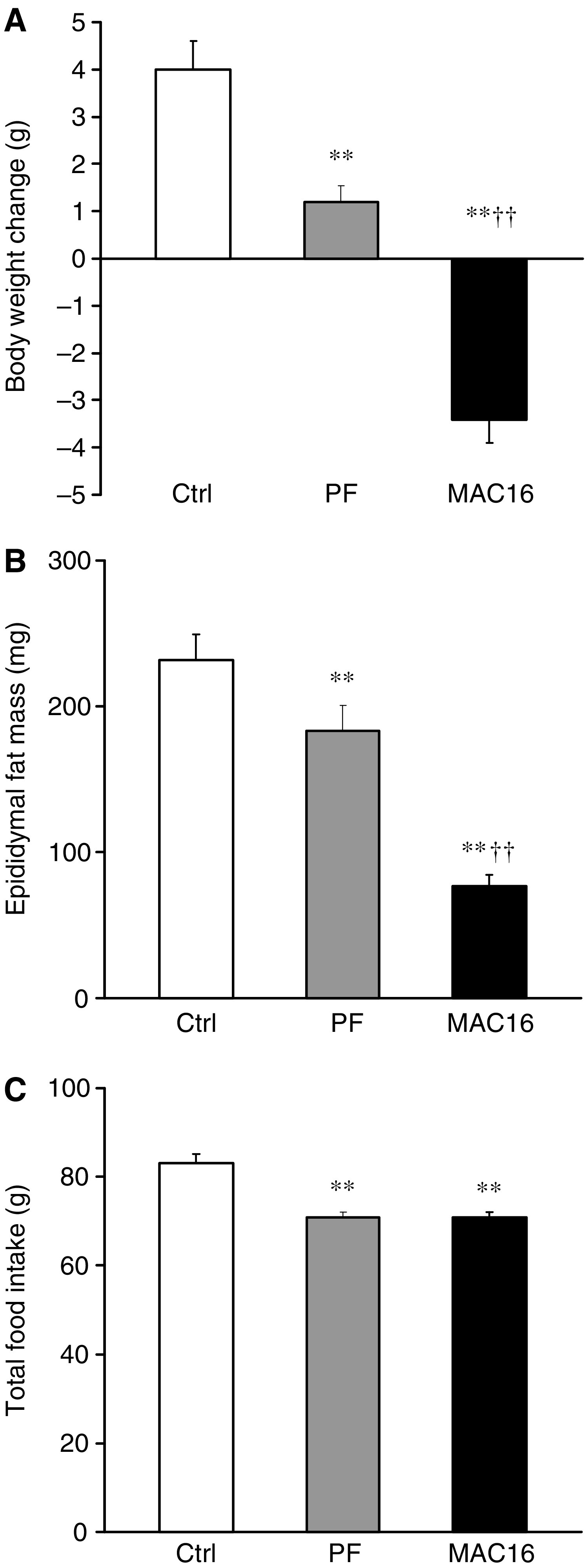
Reduced adiposity in MAC16 tumour-bearing mice. Body weight change (**A**), epididymal fat mass (**B**) and total food intake (**C**) of control (Ctrl), pair-fed (PF) and tumour-bearing mice (MAC16) at day 18 after tumour inoculation. Values are mean ±s.e.m. for eight animals per group. ^**^P<0.01 *vs* controls; ^††^*P*<0.01 *vs* pair-fed.

Tumour-bearing mice had considerably reduced body weight compared with freely fed and pair-fed controls (both *P*<0.01), and their final body weight was significantly lower than initial weight (Figure 1A). Pair-fed animals did not lose weight although their body weight was lower relative to controls (*P*<0.01) (Figure 1A). Fat mass was also markedly reduced in tumour-bearing mice while it was moderately decreased in pair-fed animals when compared with controls (−67% and −21%, both *P*<0.01) (Figure 1B). Total food intake was mildly reduced (−14%) in tumour-bearing mice relative to controls (*P*<0.01) (Figure 1C). These results confirm that the tumour-burden induces much greater reductions in body weight and fat mass than that of food restriction, demonstrating a strong cachectic effect of the MAC16 tumour on adiposity.

**Figure 2:**
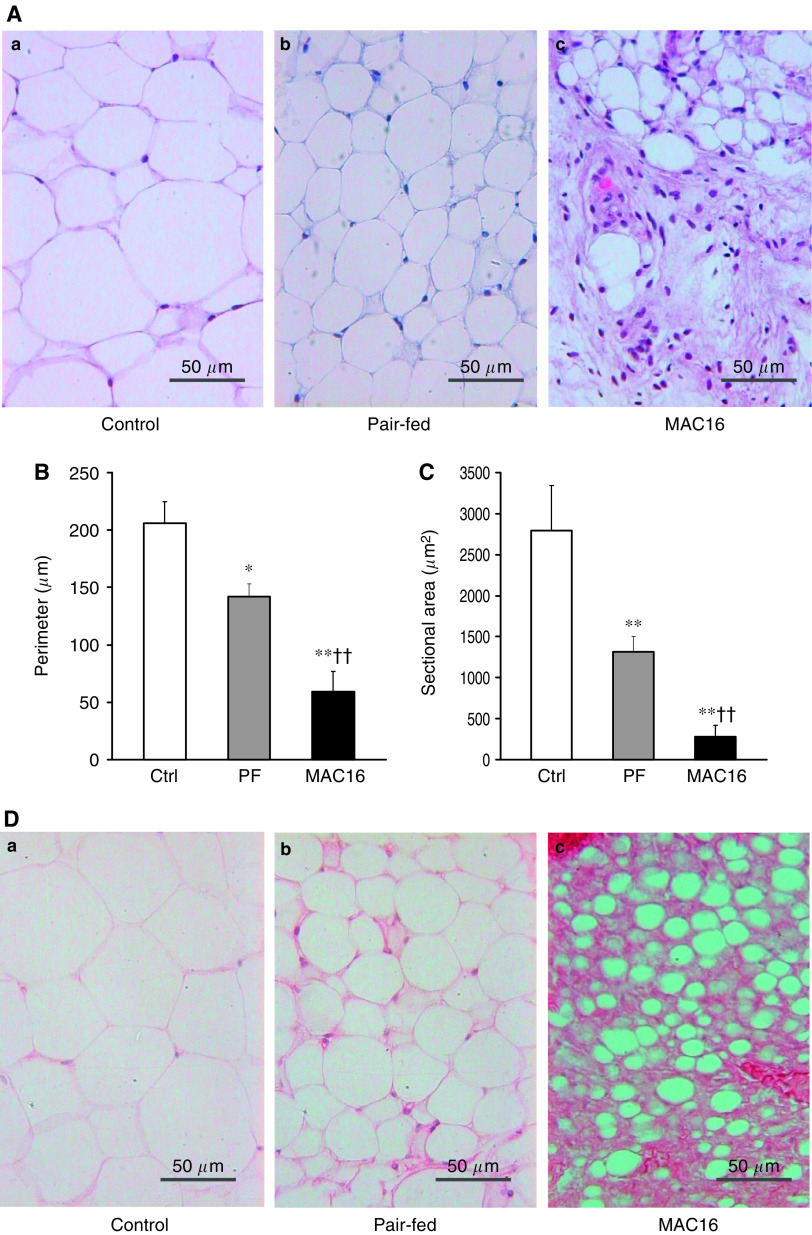
Morphological characteristics of adipose tissue. (**A**) Haematoxylin and eosin stained sections of epididymal adipose tissue from control (a), pair-fed (b) and tumour-bearing (c) mice, by light microscopy. Note many shapeless and shrunken adipocytes are present in the tissue of a tumour-bearing mouse at day 18 after tumour inoculation. Morphometric analysis of perimeter (**B**) and sectional area (**C**) of adipocytes of epididymal adipose tissue from control, pair-fed and tumour-bearing mice. Values are mean ±s.e.m. for five animals per group. ^*^P<0.05, ^**^P<0.01 *vs* controls; ^††^*P*<0.01 *vs* pair-fed. (**D**) Light microscopy of Sirius Red stained sections of epididymal adipose tissue from control (a), pair-fed (b) and tumour-bearing (c) mice at day 18 after tumour inoculation. Marked increase in collagen fibre staining is seen in the interstitial matrix of adipose tissue from tumour-bearing mice.

Examination under light microscopy has shown substantial morphological alterations of adipose tissue in tumour-bearing mice compared with freely fed and pair-fed controls. This was characterised by the tissue containing shrunken adipocytes of various sizes with a dilated interstitial space (Figure 2Aa–c). In lipoatrophic areas most adipocytes displayed irregular cell outlines (Figure 2Ac). There was no apparent infiltration of monocytes observed in adipose tissue (Figure 2Ac). Further morphometric analysis has revealed that the adipocyte size, determined as cell perimeter (Figure 2B) and sectional area (Figure 2C), was dramatically reduced in tumour-bearing mice, compared with freely fed and pair-fed controls (all *P*<0.01). To examine the nature of extracellular matrix, sections of white fat were stained with Sirius Red. There was a strong Sirius Red staining in the stroma of white fat from tumour-bearing mice, indicating a remarkable increase in collagen-fibril content in adipose tissue. However, fat cells retained a spherical appearance with only slightly increased collagen-fibril staining in the tissue matrix in pair-fed mice (Figure 2Da–c).

**Figure 3:**
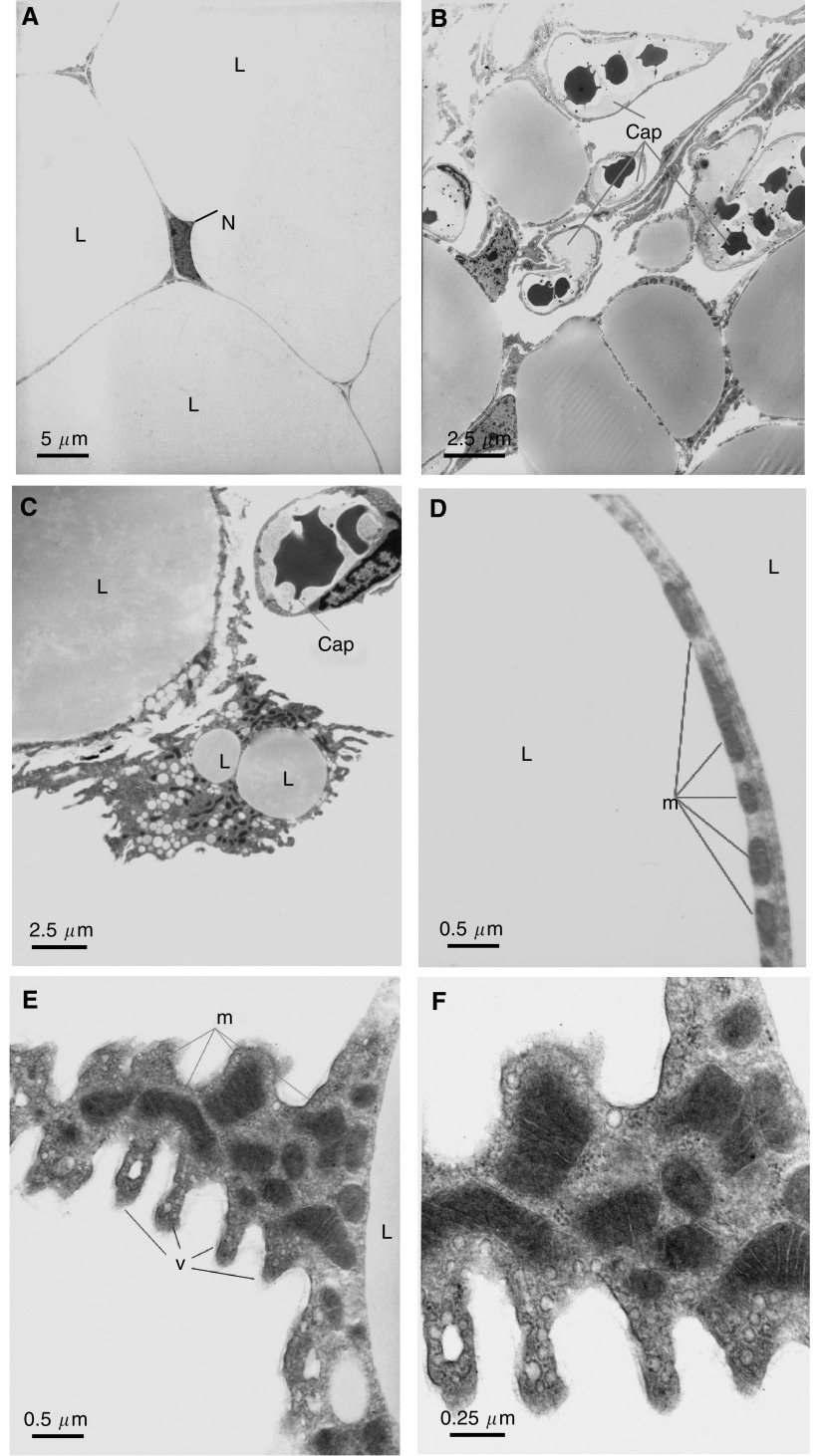
Ultrastructure of adipocytes obtained from epididymal adipose tissue. Representative electron micrograph shows adipocytes from a control mouse containing a large lipid droplet and a thin rim of cytoplasm (**A**). Adipocytes from a tumour-bearing mouse show marked reductions in size with dilated interadipocyte space (**B**). Adipocytes from a tumour-bearing mouse showing thickened peripheral rim of cytoplasm that is mitochondria-rich and contains multiple small lipid droplets (**C**). Cell membrane aspect of adipocyte from a control mouse (**D**). Adipocyte from a tumour-bearing mouse exhibits irregular cell membrane projections (villous extroflexions) with cytoplasm containing enlarged and electron dense mitochondria (**E**). Higher magnification of the same cell shows mitochondria with increased cristae and dense inner matrix (**F**). L, lipid droplet; Cap, capillary; N, nucleus; m, mitochondrion; v, villous extroflexions.

To further analyse the ultrastructural features of ‘slimmed’ adipocytes, transmission electron microscopy was performed using sections of epididymal white fat from tumour-bearing mice and freely fed controls. In agreement with what was observed by light microscopy, adipocytes from control mice were characterised by the cell containing a large lipid droplet with a thin peripheral rim of cytoplasm and the peripherally located nucleus (Figure 3A). In contrast, adipocytes from tumour-bearing mice were much smaller with dilated interadipocyte space, which was occupied by capillary vessels, while the nucleus was no longer being pressed by the large lipid droplet (Figure 3B). The peripheral rim of cytoplasm of the ‘slimmed’ adipocyte became thickened, containing numerous lipid droplets that were surrounded by mitochondria (Figure 3C). Furthermore, the mitochondria differed from typical white adipocyte mitochondria (D), were electron dense (Figure 3E) with increased cristae (Figure 3F). In addition, adipocytes exhibited irregular cytoplasmic projections and the cell surface was rich in small vacuoles (Figure 3E–F). These results illustrate an adipocyte remodelling in mice bearing the MAC16 tumour.

**Figure 4:**
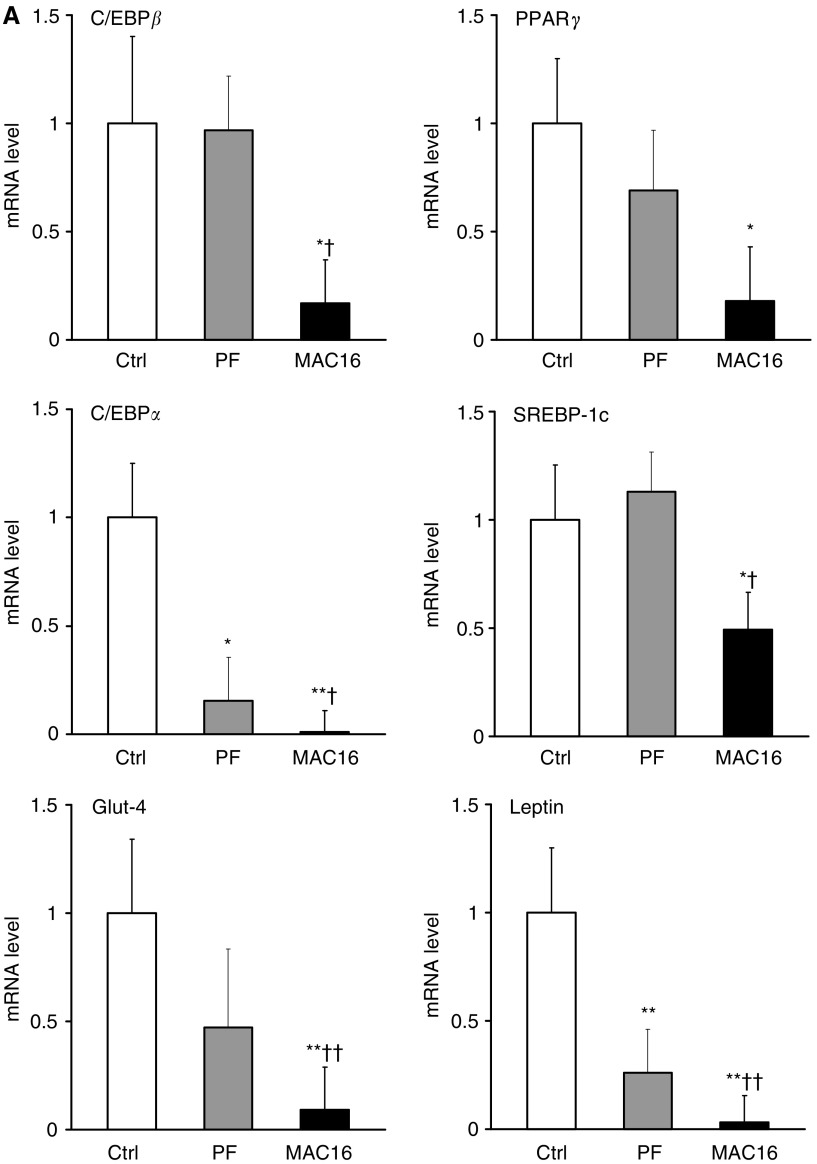

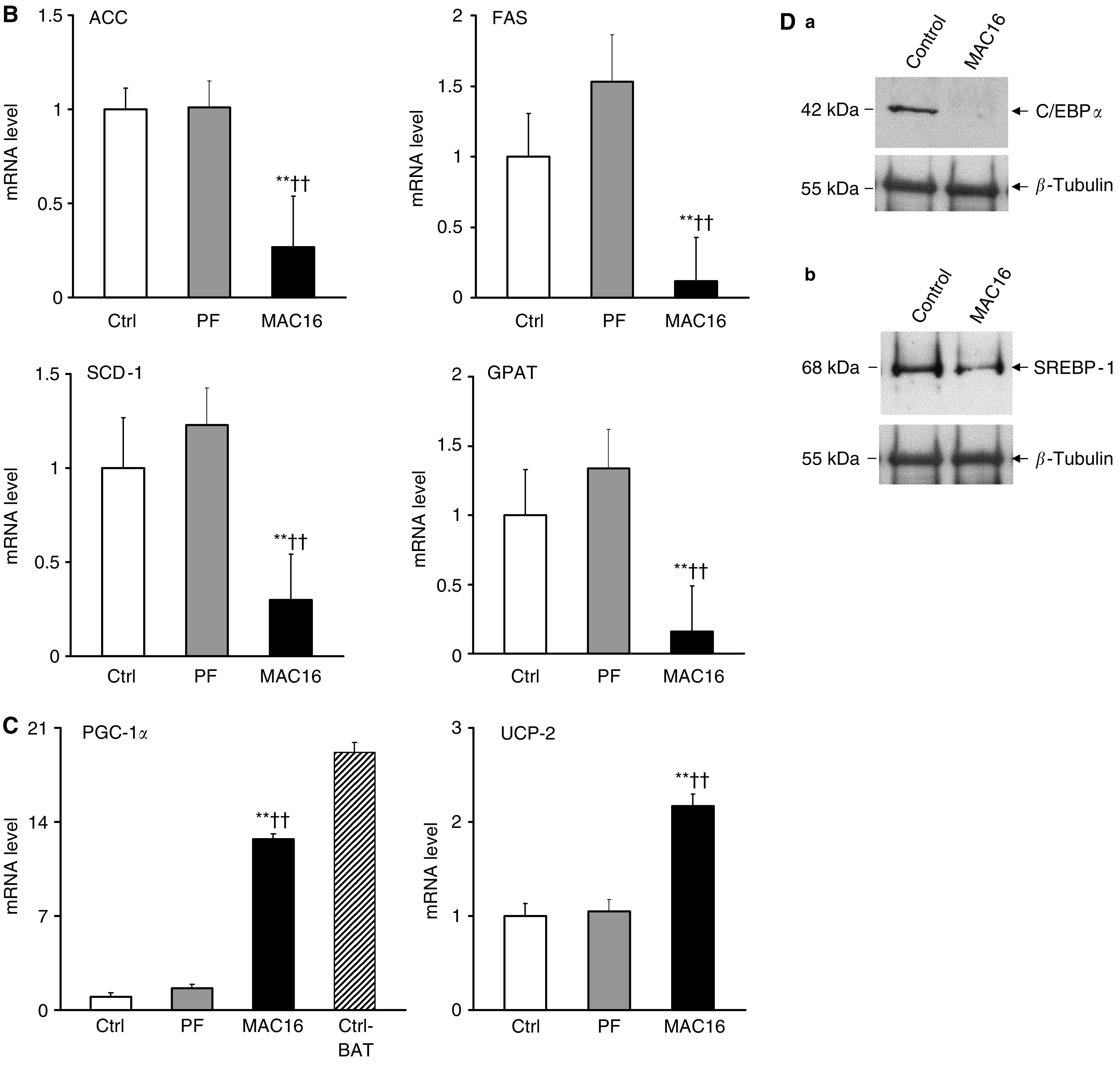
Expression levels of genes involved in adipogenesis (**A**), lipid accumulation (**B**) and lipid utilisation (**C**) in white fat. Real-time PCR analysis of RNA isolated from epididymal adipose tissue of control, pair-fed and tumour-bearing mice was performed. mRNA levels of target genes were normalised to *β*-actin. Values are mean ±s.e.m. presented as fold changes relative to controls for eight animals per group. ^*^P<0.05, ^**^P<0.01 *vs* controls; ^†^*P*<0.05, ^††^*P*<0.01 *vs* pair-fed. (**D**) Western blotting analysis of protein extracts from epididymal adipose tissue. Representative blots for C/EBP*α* and *β*-tubulin proteins (a), and for SREBP-1 and *β*-tubulin proteins (b).

To explore the molecular mechanisms by which the tumour-burden reduces fat mass, we examined the mRNA abundance of several key adipogenic factors. mRNA levels of c/EBP*β*, PPAR*γ*, c/EBP*α* and SREBP-1c fell significantly in tumour-bearing mice and there was a striking 100-fold reduction in c/EBP*α* (*P*<0.01) compared with freely fed controls (Figure 4A). Pair-feeding did not affect expression of c/EBP*β*, PPAR*γ* and SREBP-1c while a moderate decrease in c/EBP*α* mRNA (by six-fold, *P*<0.05) was observed relative to controls. In addition, mRNA levels of Glut-4 were 10-fold lower (*P*<0.01) in tumour-bearing mice than in freely fed controls, whereas pair-feeding had little effect on Glut-4 (Figure 4A). Leptin mRNA levels were dramatically decreased (by 33-fold, *P*<0.01) in tumour-bearing mice, but were only moderately reduced (by four-fold, *P*<0.01) in pair-fed mice as compared with controls (Figure 4A).

To examine whether the MAC16 tumour-burden affects the lipid storing ability of adipose tissue through inhibiting fatty acid and triglyceride synthesis, we compared the expression of SREBP-1c target – genes encoding lipogenic enzymes, ACC, FAS, SCD-1 and GPAT. As shown in Figure 4B, mRNA levels of all four enzymes were significantly decreased by at least three-fold in tumour-bearing mice (all *P*<0.01) with FAS mRNA showing a 10-fold reduction, relative to controls. However, mRNA levels of the lipogenic enzymes examined were unaffected in pair-fed animals (Figure 4B).

To assess whether the expression of genes involved in regulating lipid utilisation is altered in white fat of cachectic mice, we measured mRNA levels of PGC-1*α* and UCP-2. As shown in Figure 4C, low amounts of PGC-1*α* mRNA were detected in white fat from freely fed controls as the abundance was only ∼5% of its basal expression levels present in BAT. However, PGC-1*α* mRNA levels were dramatically increased (by 13-fold, *P*<0.01) in white fat of tumour-bearing mice, being 68% of the basal levels detected in BAT. In contrast, PGC-1*α* mRNA was unaffected in pair-fed animals. UCP-2 mRNA was elevated by over two-fold (*P*<0.01) in tumour-bearing mice, but no changes were found in pair-fed animals, relative to controls (Figure 4C).

To further examine whether the MAC16 tumour-induced inhibition in gene transcription of adipogenic factors affects their protein synthesis, the protein abundance of C/EBP*α* and SREBP-1c in white fat was examined. The tumour-burden led to suppression of C/EBP*α* protein (Figure 4D). By using an antibody that detects both SREBP-1a and -1c, the latter being the major form present in adipose tissue, a decrease in SREBP-1c protein content was observed in tumour-bearing mice compared with controls (Figure 4D).

**Figure 5:**
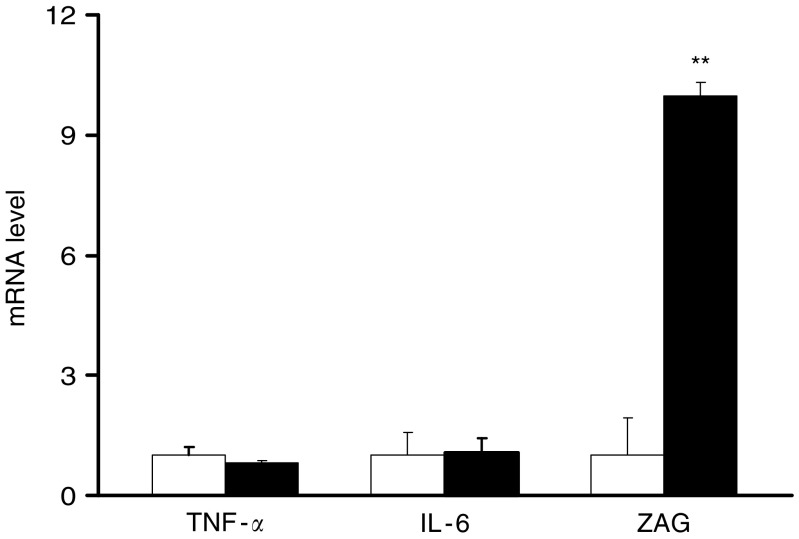
mRNA levels of TNF-*α*, IL-6 and ZAG in white fat. Real-time PCR analysis of RNA isolated from epididymal adipose tissue of control (open bars) and tumour-bearing (black bars) mice was performed. mRNA levels of target genes were normalised to *β*-actin. Values are mean ±s.e.m. presented as fold changes relative to controls; *n*=8 per group. ^**^P<0.01 *vs* control.

In order to explore whether adipose tissue-derived cytokines and lipid mobilising factor are involved in MAC16-induced lipoatrophy, gene expression of TNF*α*, IL-6 and zinc-*α*_2_-glycoprotein (ZAG) known as a lipid-mobilising factor, in white fat was determined in tumour-bearing and freely fed animals. mRNA levels of TNFα and IL-6 were unchanged in tumour-bearing mice compared with controls (both *P*>0.05) (Figure 5). As macrophage (MAC) infiltration in adipose tissue is suggested to contribute to increased local production of cytokines (Machado *et al*, 2004), we have also determined mRNA levels of two MAC makers, MAC1 and F4/80. Both were unaffected with the MAC16 tumour-burden relative to controls (MAC1, 0.99±0.37 *vs* 1.00±0.30; F4/80, 0.79±0.41 *vs* 1.00±0.40; both *P*>0.05, *n*=8 per group). In contrast, there was a 10-fold elevation in ZAG mRNA in tumour-bearing animals (*P*<0.01 *vs* controls) (Figure 5).

## DISCUSSION

The present work demonstrates profound changes at the morphological and molecular levels in adipose tissue of mice with cancer cachexia. These alterations differ greatly from that of food restriction, and are mainly characterised by adipose atrophy with marked reduction in adipocyte size and significantly increased fibrosis in the tissue matrix. In contrast, adipose tissue from pair-fed animals contain adipocytes that are spherical with only a moderate decrease in cell size and slightly increased collagen fibrils. These results illustrate that the striking alterations in adipose tissue morphology are specific to cancer cachexia in which catabolism overrides anabolic processes. Ultrastructural analysis of ‘slimmed’ adipocytes further reveals severe delipidation and modifications in cell membrane conformation and mitochondrial structure, consistent with an adipocyte remodelling under the cachectic state.

Adipocyte differentiation is orchestrated by a network of transcription factors, which are activated in a signalling cascade allowing the expression of adipocyte-specific genes that produce the differentiated phenotype (Mandrup & Lane, 1997). The present study has demonstrated for the first time substantial fall in gene transcription of the key adipogenic factors, C/EBP*β*, C/EBP*α*, PPAR*γ* and SREBP-1c, in white fat of cancer cachectic mice. Most striking of these changes was the major reduction in mRNA levels of C/EBP*α* (by 100-fold) and concomitantly its protein expression being suppressed. The importance of C/EBP*α* in adipogenesis has been shown from studies where C/EBP*α*-deficiency in mice leads to greatly reduced body fat (Wang *et al*, 1995), and C/EBP*α*-deficient adipocytes accumulate less lipid, cannot induce PPAR*γ* expression and display a complete absence of insulin-stimulated glucose transport (Wu *et al*, 1999). Our observations suggest that markedly decreased expression of adipogenic transcription factors would disrupt new adipocyte recruitment and maintaining adipocyte phenotype in cancer cachexia.

The presence of adipogenic defects in adipose tissue in cachexia is further supported by the dramatic reduction in mRNA levels of leptin, a mature adipocyte marker. This is consistent with our previous reports (Bing *et al*, 2001, 2004) and the studies using other rodent models of cancer cachexia (Lopez-Soriano *et al*, 1997b; Machado *et al*, 2004). We have also observed a decrease in Glut-4 mRNA in white fat of cachectic mice, which suggests altered glucose metabolism. Indeed, glucose metabolic rate in white fat has been shown to be decreased in mice treated with a tumour-derived lipid-mobilising factor (Russell & Tisdale, 2002). Downregulation of Glut-4 activity and protein in fat cells has also been reported in tumour-bearing rats and this might lead to reduced insulin sensitivity in adipocytes (Yoshikawa *et al*, 1997). Moreover, reduced Glut-4 expression could be a downstream effect of inhibited C/EBP*α* since the absence of this factor is associated with abnormal subcellular localisation of Glut-4 (Rosen, 2005).

The present study has shown that SREBP-1c mRNA and protein were significantly decreased in white fat of cachectic animals. SREBP-1c is known to promote lipogenesis through transcriptional regulation of fatty acid biosynthetic enzymes (Horton, 2002). Repression of SREBP-1c and its targets (ACC, FAS, SCD-1 and GPAT) in the pathway of fatty acid and triglyceride synthesis may indicate an inhibition of *de novo* lipogenesis in adipose tissue in cancer cachexia. Furthermore, reduced lipogenesis would result in a decreased amount of malonyl CoA, the product of ACC activity and an inhibitor of mitochondrial *β*-oxidation, which would be expected to remove an inhibitory regulator of fatty acid utilisation.

A growing body of evidence suggests a key role for PGC-1*α* in the coordination of mitochondrial biogenesis, and its overexpression stimulates mitochondrial proliferation (Puigserver *et al*, 1998). We have observed a major induction of PGC-1*α* mRNA in adipose tissue of tumour-bearing mice. Normally, PGC-1*α* is present in white fat at very low levels, as seen in this study, being only 5% of its basal levels in BAT. However, it rose considerably (by 13-fold) in white fat of mice with cancer cachexia. This result provides evidence that low levels of PGC-1*α* transcripts in white fat can be induced in cancer cachexia and its upregulation may indicate altered mitochondrial activity in fat cells. The present study has also shown an increase in mRNA levels of UCP-2, which is implicated in fatty acid utilisation and in reducing the generation of reactive oxygen species (Thompson & Kim, 2004). Moreover, we have observed ultrastructural changes of adipocytes from adipose tissue of cachectic mice. These ‘slimmed’ adipocytes often contained multiple lipid droplets surrounded by increased mass of mitochondria which were electron dense. Collectively, these findings suggest that adipose atrophy could be associated with alterations in mitochondrial plasticity and activity in cancer cachexia. Further studies are required to establish functional changes of mitochondria in adipose tissue wasting.

The potential mediators of lipoatrophy in cachexia are generally considered to be proinflammatory cytokines and lipid mobilising factors. TNF*α* has been shown to affect adipose tissue formation by inhibiting the differentiation of new adipocytes, causing dedifferentiation of mature fat cells and suppressing the expression of genes encoding key lipogenic enzymes (Zhang *et al*, 1996). IL-6 alone cannot inhibit preadipocyte differentiation *in vitro* (Simons *et al*, 2005) while increased mRNA levels of IL-6 and TNF*α* as well as increased MAC infiltration in adipose tissue have been reported in patients with HIV-related lipodystrophy under antiretroviral therapy (Jan *et al*, 2004). However, our data do not support a major role for these cytokines in the mediation of adipose atrophy in MAC16-induced cachexia. In our study, gene transcription of TNF*α* and IL-6 in white fat was not affected with tumour burden, in agreement with our previous report that their circulating levels were unchanged in cachectic mice (Bing *et al*, 2000). Moreover, we have illustrated morphologically no apparent monocyte infiltration in white fat, in parallel to the absence of an increase in mRNA levles of the MAC markers. Interestingly, our recent work suggests that a lipid-mobilising factor, ZAG overproduced by certain malignant tumours including the MAC16 (Todorov *et al*, 1998; Hale *et al*, 2001) and also expressed by white fat (Bing *et al*, 2004), might be responsible. In the present study, we have shown a marked increase in ZAG mRNA in white fat of cachectic mice, consistent with the previous report which has also shown an upregulation of its protein content in the tissue (Bing *et al*, 2004), suggesting that ZAG may act locally as well as at a distance to influence adipocyte metabolism. Further work is needed to establish whether ZAG has a major role in adipose tissue formation and function.

The work presented in this study demonstrates pronounced morphologic and molecular alterations of white fat in tumour-bearing animals, which indicates a strong cachectic effect of the MAC16 tumour on adipose tissue preventing lipid storage. The downregulation of the key adipogenic factors (C/EBP*β*, C/EBP*α*, PPAR*γ* and SREBP-1c) together with the repression of lipogenic factors (ACC, FAS, SCD-1, GPAT and Glut-4) suggests that impairment in the formation and lipid storing capacity of adipose tissue occurs in cancer cachexia.

## Figures and Tables

**Table 1:** Sequences of primer/probe sets used in real-time RT–PCR (TaqMan) analyses (5′ → 3′)

	**Accession no.**	**Forward/reverse primers**	**Probe**
C/EBP*α*	NM_007678	AGAGCCGAGATAAAGCCAAACA CGGTCATTGTCACTGGTCAACT	AACGTGGAGACGCAACAGAAGGTGCT
C/EBP*β*	NM_009883	ACAAGCTGAGCGACGAGTACAA TGTGCTGCGTCTCCAGGTT	CGCGAGCGCAACAACATCGC
PPAR*γ*	AY243585	AGTGGAGACCGCCCAGG GCAGCAGGTTGTCTTGGATGT	TTGCTGAACGTGAAGCCCATCGAG
Glut-4	AB008453	CATCCCACAAGGCACCCTC CATGCCACCCACAGAGAAGA	CTACGCTCTGGGCTCTCTCCGTGG
Leptin	NM_008493	CATCTGCTGGCCTTCTCCAA ATCCAGGCTCTCTGGCTTCTG	AGCTGCTCCCTGCCTCAGACCAGTG
SREBP-1c	NM_011480	GGCACTAAGTGCCCTCAACCT GCCACATAGATCTCTGCCAGTGT	TGCGCAGGAGATGCTATCTCCA
ACC	NM_133366	CCCAGCAGAATAAAGCTACTTTGG TCCTTTTGTGCAACTAGGAACGT	TGAGCATGGCATCCGGCGACT
FAS	BC046513	CCTGGATAGCATTCCGAACCT AGCACATCTCGAAGGCTACACA	CCTGAGGGACCCTACCGCATAGC
SCD-1	AF509567	TCACGACCCCACCTATCAGG TTCCTCCAGACGTACTCCAGC	TGAGGAGGGACCCCCGCCC
GPAT	MUSG3PAT	CAACACCATCCCCGACATC GTGACCTTCGATTATGCGATCA	TCGTCATACCCGTGGGCATCTCG
PGC-1*α*	XM-126624	GATGGCACGCAGCCCTAT CTCGACACGGAGAGTTAAAGGAA	CATTGTTCGATGTGTCGCCTTCTTGCT
UCP2	U69135	AAAGATACTCTCCTGAAAGCCAACCT GGCCCCGAAGGCAGAA	ATGACAGATGACCTCCCTTGCCACTTCA
TNF-*α*	NM_013693	CCCAGACCCTCACACTCAGATC GCCACTCCAGCTGCTCCTC	TAGCCCACGTCGTAGCAAACCACCAAG
IL-6	NM_031168	ACAACCACGGCCTTCCCTACTT CACGATTTCCCAGAGAACATGTG	TCACAGAGGATACCACTCCCAACAGACCT
MAC-1	X07640	GAATGGATTGTGCTATTTGTTCGG CGGAGCCATCAATCAAGAAGAC	TCCAACCTGCTGAGGCCGCCC
F4/80	X93328	AAGACTTGATACTCCAAAGTGAGC GAAGGAAGCATAACCAAGATCCC	CCCTGCACTGCTTGGCATTGCTGT
ZAG	D21059	GAGCCTGTGGGACCTTGGA CCTCCCTGGCCCTCTGAA	AATGGAGGACTGGGAGAAGGAAAGCCAG
*β*-Actin	MMU89400	ACGGCCAGGTCATCACTATTG CAAGAAGGAAGGCTGGAAAAGA	ACGAGCGGTTCCGATGCCCTG
